# Effect of Gd_2_O_3_ Addition on the Microstructure and Properties of Gd_2_O_3_-Yb_2_O_3_-Y_2_O_3_-ZrO_2_ (GYYZO) Ceramics

**DOI:** 10.3390/ma14237470

**Published:** 2021-12-06

**Authors:** Pei-Hu Gao, Sheng-Cong Zeng, Can Jin, Bo Zhang, Bai-Yang Chen, Zhong Yang, Yong-Chun Guo, Min-Xian Liang, Jian-Ping Li, Quan-Ping Li, Yong-Qing Lu, Lu Jia, Dan Zhao

**Affiliations:** 1 School of Materials and Chemical Engineering, Xi’an Technological University, Xi’an 710021, China; Z18889918364@163.com (S.-C.Z.); jincan6955@163.com (C.J.); bozhang@163.com (B.Z.); baiyang2578@163.com (B.-Y.C.); yz750925@163.com (Z.Y.); yc_guo@163.com (Y.-C.G.); lingmx@163.com (M.-X.L.); 2 Shaanxi Province Engineering Research Centre of Aluminum/Magnesium Light Alloy and Composites, Xi’an 710021, China; 3 Shanxi Disel Engine Co., Ltd., Datong 035600, China; liqp@163.com (Q.-P.L.); luyq@163.com (Y.-Q.L.); jialu@163.com (L.J.); zhaodan@163.com (D.Z.)

**Keywords:** ceramics, doping, thermal conductivity, hardness, modulus, fracture toughness

## Abstract

Gd and Yb elements have high chemical stability, which can stabilize the solid solution in ZrO_2_. Gd_2_O_3_ and Yb_2_O_3_ have high melting points, and good oxidation resistance in extreme environments, stable chemical properties. Therefore, Gd_2_O_3_ and Yb_2_O_3_ were added to ZrO_2_ to stabilize oxides, improve the high temperature stability, and effectively decrease the thermal conductivity at high temperature. In this work, 5 wt% Yb_2_O_3_ and 5 wt%, 10 wt%, 15 wt% Gd_2_O_3_ were doped into 8 wt% Y_2_O_3_ stabilized ZrO_2_ (8YSZ) powders as thermal barrier coating materials, and sintered at 1650 °C for 6 h, 12 h, 24 h. The effects of Gd_2_O_3_ addition on the microstructure, density, thermal conductivity, hardness, and fracture toughness of Gd_2_O_3_-Yb_2_O_3_-Y_2_O_3_-ZrO_2_ (GYYZO) bulk composite ceramics were investigated. It was found that the densification of the 8YSZ bulk and GYYZO bulk with 15 wt% Gd_2_O_3_ reached 96.89% and 96.22% sintered at 1650 °C for 24 h. With the increase of Gd_2_O_3_ addition, the hardness, elastic modulus and fracture toughness of the GYYZO bulk increased and the thermal conductivity and thermal expansion coefficient of the GYYZO bulk decreased. GYYZO bulk with 15 wt% Gd_2_O_3_ sintered at 1650 °C for 24h had the highest hardness, elastic modulus and fracture toughness of 15.61 GPa, 306.88 GPa, 7.822 MPa·m^0.5^, and the lowest thermal conductivity and thermal expansion coefficient of 1.04 W/(m·k) and 7.89 × 10^−6^/°C at 1100 °C, respectively. The addition of Gd_2_O_3_ into YSZ could not only effectively reduce the thermal conductivity but also improve the mechanical properties, which would improve the thermal barrier coatings’ performances further.

## 1. Introduction

The research, development, and manufacturing of gas turbines and aero engines are important standards of measuring the manufacturing level of a country’s advanced industries [1]. At present, the combustion temperature of advanced gas turbines has exceeded 1500 °C, which means that the high-temperature components of gas turbines will face a more severe service environment [2]. Thermal barrier coatings have been widely used in hot sections of advanced gas turbines and aeroengines to improve the reliability and durability as well as the efficiency of engines [3]. The often-used thermal barrier coatings compose of a thermally insulating ceramic layer with a high melting point and a thickness of about 300–500 μm as well as low thermal conductivity and an oxidation-resistant bonding layer. The thermal barrier coating isolates the contact between the high-temperature working medium and the metal substrate. TBCs’ good heat insulation performance reduces the surface temperature of the parts, weakens the heat exchange effect, and forms an effective protection for the substrate [4,5,6].

The preparation methods of thermal barrier coatings mainly include electron beam physical vapor deposition and plasma spraying [7,8]. TBCs prepared through plasma spraying have typical lamellar structural features [9]. During the serving of TBCs at high temperature, perforation cracks, or cracks parallel to the surface of the coating will form in the ceramic coatings or near the boundary between the ceramic coating and bond coating [10,11]. Cracks parallel to the substrate surface easily lead to cracking failure of the coating, which is very harmful to thermal shock resistance of TBCs. The compatibility can be improved by adding a bonding layer between the substrate and the ceramic layer. Meanwhile, TBCs prepared through the electron beam physical vapor deposition method have columnar characters which will lead to higher thermal conductivity than those prepared by plasma spraying and finally weaken the thermal isolation effects [12,13].

Studies have proposed that changing the composition of the thermal barrier coating material can achieve lower thermal conductivity and better high temperature resistance than often-used ceramic materials [14]. The use of co-doped trivalent or pentavalent rare earth oxides into zirconia ceramic materials can change the structure of the thermal barrier coatings [15]. Rare earth elements have special electronic layer structure and activity, low electronegativity, and special chemical activity [16]. In addition, rare earth elements have strong adsorption capacity for other elements, high chemical stability, and high melting point, and can be dissolved in a limited solid solution of ZrO_2_. Therefore, the addition of rare earth elements can greatly improve the performance of the material [17,18]. Stabilizers, such as Y_2_O_3_, MgO, CeO_2_, and CaO, are used to hinder phase changes of Zirconia at high temperature. Y_2_O_3_ is thought to be the preferred stabilizer with the content of 6–12 wt%. In particular, 8 mol-YSZ has the stabilized cubic structure. Meanwhile, the thermal conductivity of 8 mol-YSZ is 2.3 W/(m·k) at room temperature and 1.85 W/(m·k) at 1000 °C [19]. However, when the temperature is above 1200 °C, sintering and phase change will occur. Rare earth elements are strongly oxygen-philic elements. Rare earth oxides are the most stable compounds [20,21]. In recent years, pyrochlore structure (A_2_B_2_O_7_) is considered to have good prospects in thermal barrier coating materials with low thermal conductivity, high melting point, good phase stability at high temperature and a large thermal expansion coefficient [22,23,24,25]. Gd_2_O_3_ and Yb_2_O_3_ have high melting points of 2350 °C and 2346 °C, respectively. Meanwhile, both of them have good oxidation resistance, stable chemical activity, and good impact resistance in extreme environments [26]. Oxide co-dopped yttria stabilized zirconia ceramic material can change the structure of the thermal barrier coating system to make it a stable structure. In addition, Gd and Yb elements have a strong adsorption capacity for other elements, high chemical stability, and can be dissolved in a limited solid solution of ZrO_2_ [27,28,29,30]. Therefore, the addition of Gd and Yb elements may greatly improve the performance of ZrO_2_ thermal barrier coatings. 

Therefore, in this work, quaternary Gd_2_O_3_-Yb_2_O_3_-Y_2_O_3_-ZrO_2_ (GYYZO) materials were prepared to investigate the microstructure and properties including thermal conductivity, thermal expansion, hardness as well as modulus to expand the application materials in thermal barrier coatings.

## 2. Experimental Materials and Procedures

### 2.1. Preparation of Bulks

**Figure 1:**
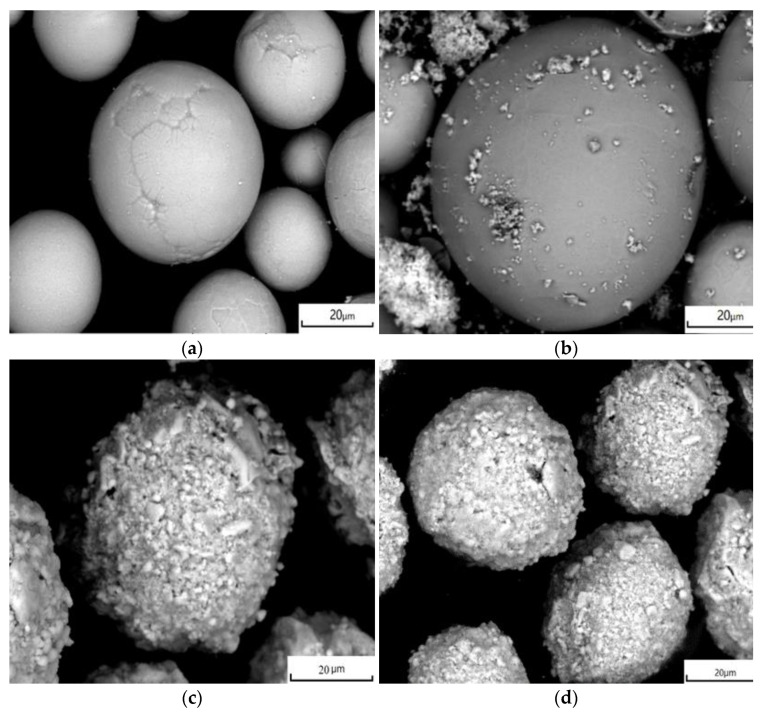
Morphology of powder: (**a**) 8YSZ; (**b**) GYYZO with 5 wt% Gd_2_O_3_ and 5 wt% Yb_2_O_3_; (**c**) GYYZO with 10 wt% Gd_2_O_3_ and 5 wt% Yb_2_O_3_; (**d**) GYYZO with 15 wt% Gd_2_O_3_ and 5 wt% Yb_2_O_3_.

In Yb_2_O_3_ stabilized ZrO_2_ (YbSZ), 5 wt% Yb_2_O_3_ stabilized ZrO_2_ had the lowest heat capacity and 10 wt% Yb_2_O_3_ stabilized ZrO_2_ had a fully stable cubic phase [31,32]. The properties of Gd_2_O_3_ and Yb_2_O_3_ were similar. Therefore, the contents of Yb_2_O_3_ and Y_2_O_3_ were set as 5 wt% and 8 wt% to keep the low heat capacity and stable tetragonal phase of the Gd_2_O_3_-Yb_2_O_3_-Y_2_O_3_-ZrO_2_ (GYYZO) composites. The influence of Gd_2_O_3_ addition on the microstructure and properties of GYYZO composite would be investigated mainly. For the convenience of preparing the composite, 5 wt% Yb_2_O_3_ powder and 5 wt%, 10 wt%, 15 wt% Gd_2_O_3_ powders were respectively added into 8YSZ powders (M204NS, Metco, Boston, MA, USA) through ball milling (ND7—04, Nanda Tianzun Electronic Co., Ltd., Nanjing, China) at a rotation speed of 120 rpm. The ball milling parameters were shown in Table 1. During the powders mixing process, the ball to powder weight ratio was controlled at 10:1. To avoid contamination, agate ball and jar were used. The powders’ mixing times were 20 h. One percent of sodium stearate was added as an additional wetting agent in weight. 8YSZ powder had a mean size of 30 µm. Both Yb_2_O_3_ and Gd_2_O_3_ powder had a mean size of 3 µm. The powder had a globular shape as shown in Figure 1. After mixing through ball milling, small Gd_2_O_3_ and Yb_2_O_3_ powders stuck to the large 8YSZ powder to form mixed powders. The mixed powders were compacted into a bulk with a diameter of 13 mm and a thickness of 3 mm through an isostatic press (ZJYP-60T, Tianjin Xinnuo Instrument Co., Ltd., Tianjin, China) under an isostatic pressure of 200 MPa for a holding time of 5 min. The detailed parameters were listed in Table 2. After cold compaction, the preform bodies were sintered at 1650 °C for 6 h, 12 h, and 24 h.

### 2.2. Microstructures and Phases

The morphology of the original powder and the microstructure of sintered bulk were examined by scanning electron microscopy (SEM; VEGA II-XMU, TESCAN, Brno, Czech Republic). The phases were analyzed through X-ray diffraction (XRD-6000, Shimadzu, Kyoto, Japan) with Cu Kα radiation with a step of 0.02° from 20° to 80° at a scanning speed of 2°/min.

### 2.3. Properties

According to the Archimedes drainage method as well as an ISO 1183-1:2004 standard, the bulk’s density was measured. A balance with an accuracy of 0.0001 g (PTY-504, Fuzhou Huazhi Scientific Instrument Co., Ltd., Fuzhou, China) was used for weighing, and the density was calculated according to Formula (1):

(1) $$\rho_{s}=\frac{m_{1}}{m_{1}-m_{2}}(\rho_{0}-\rho_{L})+\rho_{L}$$

where *ρ*_s_ was the density of the sample, *ρ*_0_ was the water density of 0.998 g/cm^3^, *ρ*_L_ was the air density of 0.0012 g/cm^3^, m_1_ was the mass of the weighed sample in air, and m_2_ was the sample weighed in water. Each sample was measured for five times and the average value was adopted.

The nanoindentation method was used to test the modulus and hardness with a nanomechanical testing system (Hysitron TI Premier, Bruker, MN, USA). The prismatic indenter was used with a load of 10 mN for the loading time, holding time, and unloading time of 5 s, 3 s, and 5 s, respectively. When the low-load indentation test is used, radial cracks can be obtained. The cracks produced in this experiment are all radial cracks, and the crack length calculation method is adopted. The relationship between fracture toughness and crack length is shown in Formula (2):

(2) $$K_{IC}=1.073\cdot \alpha \cdot {\left(\frac{E}{H}\right)}^{1/2}\cdot \left(\frac{P}{C^{3/2}}\right)$$

where P was the maximum indentation load, C was the length of the crack, and α was a coefficient related to the shape of the indenter, where 0.016 was taken, E was the modulus, and H was the hardness.

The thermal conductivity was tested through laser flash heating method with a laser thermal conductivity meter (DLF-1200, TA, New Castle, DE, USA). The value of thermal conductivity was calculated according to Formula (3):

(3) $$K=DC_{p}\rho$$

where K was the thermal conductivity (W·m^−1^·K^−1^), D was the thermal diffusivity (m^2^·s^−1^), Cp was the specific heat (J·kg^−1^·K^−1^), and ρ was the room temperature density of the sample (kg·m^−3^).

The coefficient of thermal expansion was tested with a thermal expansion meter (SDTA840, TA, New Castle, DE, USA). The coefficient of thermal expansion was calculated according to Formula (4):

(4) $$\alpha =\frac{\left(L_{T}-L_{0}\right)}{L_{0}\left(T-T_{0}\right)}$$

where α was the thermal expansion coefficient of the material, L_T_ and L_0_ were the length of the sample at the temperature of T and room temperature of T_0_, respectively.

## 3. Results

### 3.1. Microstructure

**Figure 2:**
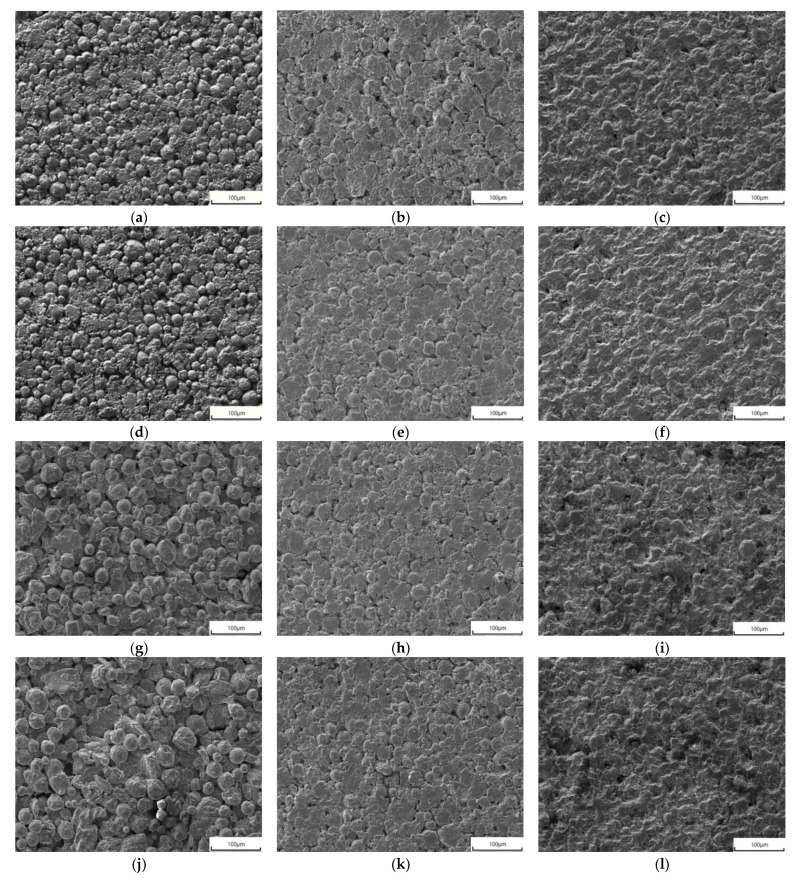
Microstructure of the sintered 8YSZ bulk at 1650 °C for 6 h (**a**), 12 h (**b**), and 24 h (**c**); microstructure of the sintered GYYZO bulk with 5 wt% Yb_2_O_3_ and 5 wt% Gd_2_O_3_ at 1650 °C for 6 h (**d**), 12 h (**e**), and 24 h (**f**); microstructure of the sintered GYYZO bulk with 5 wt% Yb_2_O_3_ and 10 wt% Gd_2_O_3_ at 1650 °C for 6 h (**g**), 12 h (**h**), and 24 h (**i**); microstructure of the sintered GYYZO bulk with 5 wt% Yb_2_O_3_ and 15 wt% Gd_2_O_3_ at 1650 °C for 6 h (**j**), 12 h (**k**), and 24 h (**l**).

Figure 2 shows the microstructure of the 8YSZ and GYYZO bulks with 5 wt% Yb_2_O_3_ and 5 wt%, 10 wt%, and 15 wt% Gd_2_O_3_ sintered at 1650 °C for 6 h, 12 h, and 24 h. With the prolongation of the sintering time at 1650 °C, the sintered 8YSZ bulk became denser and denser. The original powders’ interfaces were obvious in the sintered 8YSZ bulk at 1650 °C for 6 h as seen in Figure 2a, while the original powders’ interfaces became blurred when the sintering times reached 12 h and 24 h as seen in Figure 2b,c. The powders’ interfaces disappeared gradually with the sintering and densification of 8YSZ. With the prolongation of the sintering time at 1650 °C, the sintered GYYZO bulk with 5 wt% Yb_2_O_3_ and 5 wt% Gd_2_O_3_ became denser and denser, which was similar to the densification of 8YSZ. The original powders’ interfaces were obvious in the sintered 5 wt% Gd_2_O_3_-GYYZO bulk at 1650 °C for 6h as seen in Figure 2d. As compared to the sintered 8YSZ at 1650 °C for 6 h, the original powders’ interfaces were more obvious in the sintered 5 wt% Gd_2_O_3_-GYYZO bulk at 1650 °C for 6 h than those in 8YSZ at 1650 °C for 6 h, which reflected that 5 wt% Gd_2_O_3_-GYYZO had higher sintering resistance than 8YSZ. With the prolongation of the sintering time at 1650 °C, the original powders’ interfaces were sealed gradually in the sintered 5 wt% Gd_2_O_3_-GYYZO bulk as seen in Figure 2e,f, which was similar to the densification of 8YSZ too. When the sintering time reached 12 h as seen in Figure 2e, the original powders’ interfaces became smooth and connected with each other. When the sintering time reached 24 h as seen in Figure 2f, the original powders’ interfaces almost disappeared.

With the prolongation of the sintering time at 1650 °C, the densification of the sintered 10 wt% Gd_2_O_3_-GYYZO and 15 wt% Gd_2_O_3_-GYYZO bulks were similar to the densification of 8YSZ and 5 wt% Gd_2_O_3_-GYYZO. The original powders’ interfaces were obvious in the sintered 10 wt% Gd_2_O_3_-GYYZO and 15 wt% Gd_2_O_3_-GYYZO bulks at 1650 °C for 6 h as seen in Figure 2g,j. As compared to the sintered 8YSZ and 5 wt% Gd_2_O_3_-GYYZO bulk at 1650 °C for 6 h, the original powders’ interfaces were more obvious in the sintered 10 wt% Gd_2_O_3_-GYYZO and 15 wt% Gd_2_O_3_-GYYZO bulks at 1650 °C for 6 h than those in 8YSZ and 5 wt% Gd_2_O_3_-GYYZO at 1650 °C for 6 h, which reflected that 10 wt% Gd_2_O_3_-GYYZO and 15 wt% Gd_2_O_3_-GYYZO bulks had higher sintering resistance than 8YSZ and 5 wt% Gd_2_O_3_-GYYZO in the same sintering condition. With the increase of Gd_2_O_3_ addition, the sintering resistance of GYYZO became higher and higher. In the same sintering conditions, 15 wt% Gd_2_O_3_-GYYZO had the highest sintering resistance in the three GYYZO bulks with different contents of Gd_2_O_3_. With the prolongation of the sintering time at 1650 °C, the original powders’ interfaces were sealed gradually in the sintered 10 wt% Gd_2_O_3_-GYYZO and 15 wt% Gd_2_O_3_-GYYZO bulks, which were similar to the densification of 8YSZ, 5 wt% Gd_2_O_3_-GYYZO too. When the sintering time reached 12 h, the original powders’ interfaces became smooth and connected with each other. Meanwhile, the densification of the sintered 15 wt% Gd_2_O_3_-GYYZO, as seen in Figure 2k, was comparable to both 5 wt% Gd_2_O_3_-GYYZO and 10 wt% Gd_2_O_3_-GYYZO, as seen in Figure 2e,h, when the sintering time reached 12 h. When the sintering time reached 24 h, the original powders’ interfaces in 15 wt% Gd_2_O_3_-GYYZO bulk disappeared almost as seen in Figure 2l, which was similar to 8YSZ, 5 wt% Gd_2_O_3_-GYYZO and 10 wt% Gd_2_O_3_-GYYZO as seen in Figure 2c,f,i. Although there were differences of the original powders’ interfaces in the three GYYZO bulks sintered at 1650 °C for 6 h, all of the three GYYZO bulks with different contents of Gd_2_O_3_ could be densified thoroughly when the sintering times reached 24 h at 1650 °C.

The image processing method was used to determine the porosity of the sintered bulks through ImageJ Software^@^ with the results listed in Table 3. With the prolongation of the sintering time, the porosity of the sintered bulks gradually decreased. The bulk material sintered for 24 h had the lowest porosity. Meanwhile, with the increase of gadolinium oxide addition, the porosity of the bulk material gradually increased for that the addition of gadolinium oxide improved the sintering resistance of the GYYZO bulks. 

### 3.2. Phases

**Figure 3:**
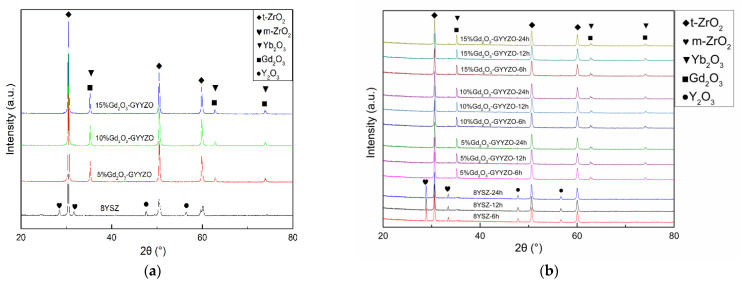
XRD analysis of powder and bulk materials: (**a**) powder; (**b**) bulk materials.

Figure 3 shows the XRD patterns of the powder and sintered bulks. In the commixed powders, the XRD pattern of 8YSZ powder had a part of monoclinic phase at 27° and 32° according to the PDF card (No. 72-0597). Meanwhile, there was no monoclinic phase but tetragonal phase (PDF card No. 71-1282) in the GYYZO powder. The addition of Gd_2_O_3_ (PDF card No. 24-0430) and Yb_2_O_3_ (PDF card No. 74-1981) could replace some part of Y_2_O_3_ (PDF card No. 20-1412) and improve the stability of 8YSZ. In the XRD pattern of the bulk material, the sintered 8YSZ bulk material had a partial monoclinic phase at 27° and 32°, and its diffraction strength was higher than that of the powder. After sintering at 1650 °C, parts of the 8YSZ experienced phase change, and more monoclinic phases formed after sintering. However, the sintered GYYZO bulk materials did not contain the monoclinic phase. The sintered GYYZO bulks maintained the same phase structure as the powder. The GYYZO bulks did not undergo a phase transition after sintering at 1650 °C. The diffraction peak strength of the monoclinic phase in the sintered 8YSZ bulk at 27° and 32° became stronger with the increase of the sintering time. The phase structures of the sintered GYYZO bulks were basically the same. The addition of Gd_2_O_3_ and Yb_2_O_3_ could improve the stability of 8YSZ.

### 3.3. Densification

Table 4 shows the actual density of the sintered bulks tested by the Archimedes drainage method. The bulks sintered at 1650 °C for 24 h had the highest density. All of the GYYZO composite bulks’ densities were higher than the sintered 8YSZ because both Gd_2_O_3_ and Yb_2_O_3_ had relative higher densities than ZrO_2_ and Y_2_O_3_.

The theoretical densities of the 8YSZ and GYYZO with 5 wt%, 10 wt% as well as 15 wt% Gd_2_O_3_ composite ceramics were 5.718 g/cm^3^, 6.022 g/cm^3^, 6.088 g/cm^3^ and 6.156 g/cm^3^, respectively. The densification degree of the bulks could be calculated by the ratio of actual density to theoretical density. Table 5 shows the densification degree of the sintered bulks in different compositions for different sintering times. The composite bulks sintered at 1650 °C for 24 h had the highest densification degree. With the increase of gadolinium oxide addition, the densification degree decreased gradually because the addition of gadolinium oxide improved the sintering resistance of the composites for gadolinium oxide’s high melting point.

### 3.4. Thermal Conductivity

**Figure 4:**
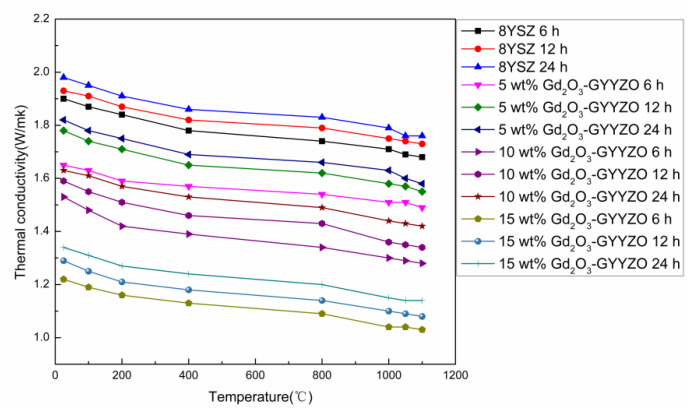
Thermal conductivities of the sintered bulk materials.

Figure 4 shows the thermal conductivities of the sintered bulks tested through the laser flash heating method at the temperatures from room temperature to 1100 °C. The thermal conductivity gradually decreased with the increase of Gd_2_O_3_ addition. The sintered GYYZO bulk with 15 wt% Gd_2_O_3_ had the lowest thermal conductivity of 1.04 W/(m k) at 1100 °C, which was much lower than that of 8YSZ with the thermal conductivity of 1.78 W/(m k) at 1100 °C and the reported 100% densified 8YSZ with the thermal conductivity of 2.3 W/(m k) at room temperature and 1.85 W/(m k) at 1000 °C [19]. The addition of Gd_2_O_3_ could reduce the thermal conductivity of YSZ further. With the prolongation of sintering times, the sintered bulks’ thermal conductivities increased gradually accompanied with the densification.

### 3.5. Thermal Expansion Coefficient

**Figure 5:**
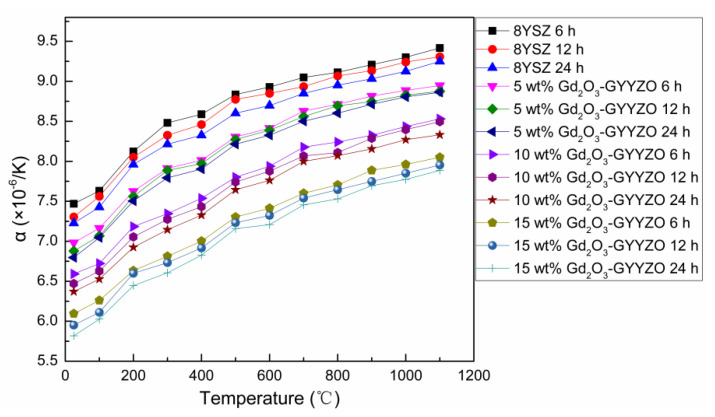
Thermal expansion coefficient of the sintered bulks.

Figure 5 shows the thermal expansion coefficients of the sintered bulks. Generally, all of the composite materials’ thermal expansion coefficients increased with the increase of the serving temperature. The thermal expansion coefficient decreased gradually with the increase of Gd_2_O_3_ addition. The sintered 15 wt% Gd_2_O_3_-GYYZO bulk with sintered at 1650 °C for 24 h had the lowest thermal expansion coefficient of 7.89 × 10^−6^/°C at 1100 °C, which was 14.7% lower than that of 8YSZ with the thermal expansion coefficient of 9.25 × 10^−6^/°C at 1100 °C. The addition of Gd_2_O_3_ could reduce the thermal expansion coefficient of the YSZ material, which could improve the high temperature stability of the material, while it might increase the gap of thermal expansion coefficients between the ceramic and the metal substrate, which would be not helpful for the direct use of GYYZO ceramics in thermal barrier coatings.

### 3.6. Mechanical Properties

**Figure 6:**
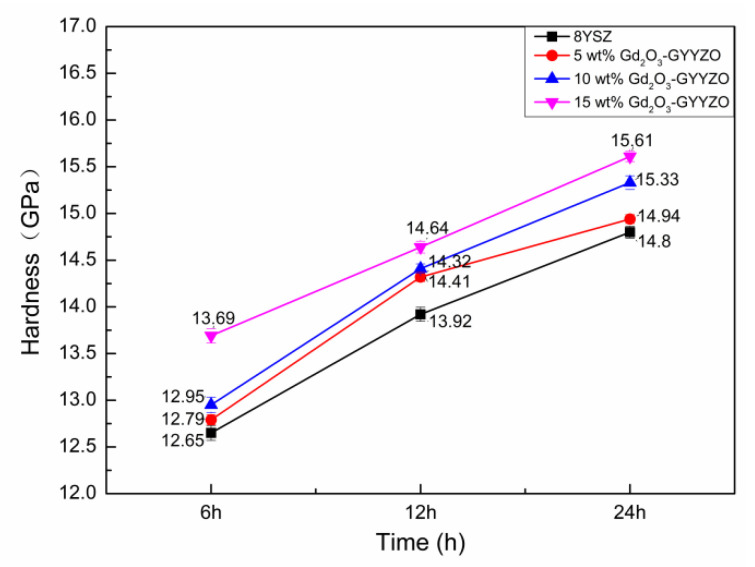
Hardness of the sintered bulks.

Figure 6 shows the hardness of the bulks sintered at 1650 °C for 6 h, 12 h, and 24 h. The sintered GYYZO bulks’ hardness increased with the increase of Gd_2_O_3_ addition and the sintering times. The 15 wt% Gd_2_O_3_-GYYZO bulk sintered at 1650 °C for 24 h had the highest hardness of 15.61 GPa, which was 5.4% higher than that of 8YSZ with the hardness of 14.8 GPa. The addition of Gd_2_O_3_ and Yb_2_O_3_ could improve the hardness of the composite material effectively. The high hardness could be attributed to two aspects. One was the densification. With the prolongation of sintering time, the densifications of the sintered GYYZO bulks were higher than 96%, which was close to full densification and could contribute to high hardness. The other was the composition’s strengthening. According to the literature, the hardness of Gd_2_O_3_ bulk was 7.9 GPa [33], the hardness of Y_2_O_3_ bulk was 6.0 GPa, and the hardness of 5YbSZ block was 12 GPa [34]. The theoretical hardness of 15 wt% Gd_2_O_3_-GYYZO can be simplified calculated to be 10.905 GPa according to the component’s hardness in 15 wt%Gd_2_O_3_-5 wt% Yb_2_O_3_-8 wt% Y_2_O_3_-ZrO_2_ (15 wt% Gd_2_O_3_-GYYZO). The tested hardness of the sintered 15 wt% Gd_2_O_3_-GYYZO bulk was 15.61 GPa. The tested hardness was 43.2% higher than the simplified calculated one, which was attributed to the good stabilization of ZrO_2_ in tetragonal structure by Gd_2_O_3_, Yb_2_O_3_, and Y_2_O_3_ as well as the solid strengthening.

**Figure 7:**
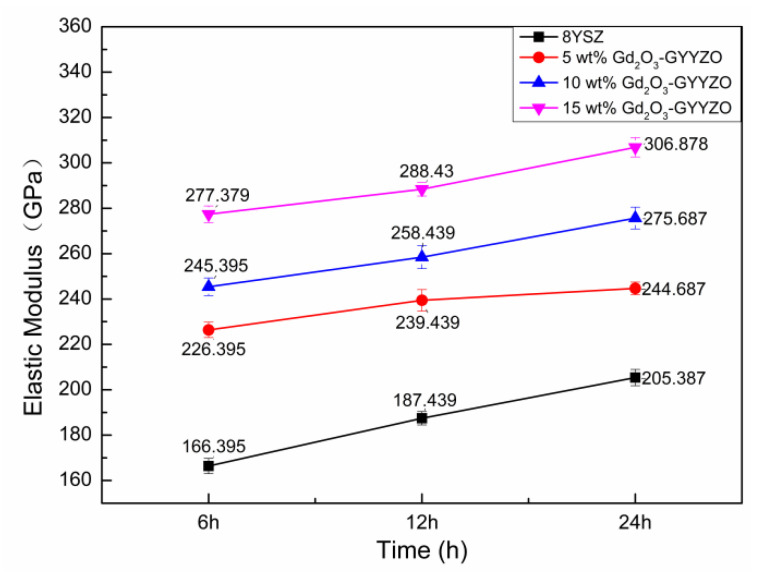
Elastic modulus of the sintered bulks.

Figure 7 shows the elastic modulus of the bulks sintered at 1650 °C for 6 h, 12 h, and 24 h. The elastic modulus of bulk materials increased with the increase of Gd_2_O_3_ addition as well as the sintering times. The 15 wt%Gd_2_O_3_-GYYZO bulk sintered at 1650 °C for 24 h had the highest elastic modulus of 306.88 GPa, which was 49.4% higher than that of the sintered 8YSZ bulk with the elastic modulus of 205.387 GPa. The addition of Gd_2_O_3_ and Yb_2_O_3_ could improve the elastic modulus of the composite materials effectively.

**Figure 8:**
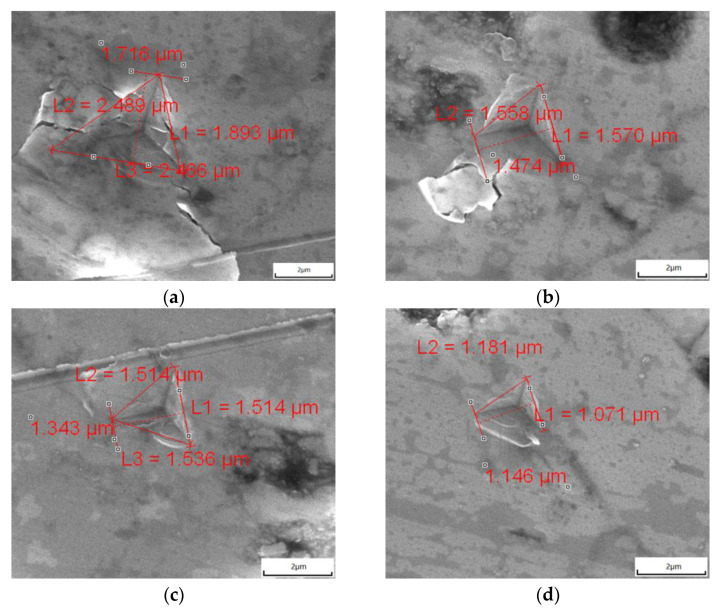
Indentations of the sintered 8YSZ bulk (**a**); GYYZO bulk with 5 wt% Gd_2_O_3_ (**b**); 10 wt% Gd_2_O_3_ (**c**) and 15 wt% Gd_2_O_3_ (**d**).

When the low-load indentation test was performed, radial cracks could be obtained. Figure 8 shows the nanoindentation with cracks in the 8YSZ, GYYZO bulks with 5 wt%, 10 wt%, and 15wt% Gd_2_O_3_ sintered at 1650 °C for 24 h. The fracture toughness values K_IC_ of the sintered bulks were calculated according to Equation (3) with the results shown in Table 6. With the increase of sintering time, all the fracture toughnesses of the sintered 8YSZ, GYYZO bulks with 5 wt%, 10 wt%, and 15 wt% Gd_2_O_3_ increased accompanying with the densification. Meanwhile, with the increase of the Gd_2_O_3_ addition, the fracture toughness of the sintered GYYZO bulk increased. When the addition of Gd_2_O_3_ reached 15 wt%, the highest fracture toughness reached 7.822 MPa·m^0.5^, which was 22.4% higher than that of the densest 8YSZ with K_IC_ value of 6.391 MPa·m^0.5^. Jan et al. [35] reported that the fracture toughness of 8YSZ was approximately 5.1 MPa·m^0.5^.

## 4. Discussion

Zirconia had a high melting point (2680 °C), low thermal conductivity, good oxidation resistance, stable chemical properties, good impact resistance, and a similar expansion coefficient to metal materials (8–10.4 × 10^−6^/°C) so that it was commonly used as a thermal barrier coating material. Meanwhile, its phase structure would change at a high temperature, resulting in volume changes (for example, monoclinic phase and tetragonal phase had different lattice parameters), stresses in the coating, and finally cracks and failures. The coating would be peeled off and fail under thermal shock conditions for the accumulation of thermal stresses induced by thermal and phase changes [36]. Therefore, stabilizers, such as MgO, CaO, Y_2_O_3_, and so on, were added to the pure ZrO_2_ to stabilize the phase structures and prevent failures of thermal barrier coatings from phase changes while serving at a high temperature. Y_2_O_3_ was the most widely used stabilizer. The thermal conductivity of 8YSZ bulk was 1.85 W/(m·K) [19], and the coefficient of thermal expansion was 10 × 10^−6^/°C, which was difficult to meet the requirements of high-performance aero-engines for high thermal insulation [37]. Gd_2_O_3_ and Yb_2_O_3_ had high melting points (2350 °C and 2346 °C, respectively), good oxidation resistance and impact resistance in extreme environments as well as stable chemical properties. In addition, Gd and Yb elements had strong adsorption capacities for other elements so that they could be dissolved in a limited solid solution in ZrO_2_ to improve the thermal barrier coatings’ performance further [38].

**Figure 9:**
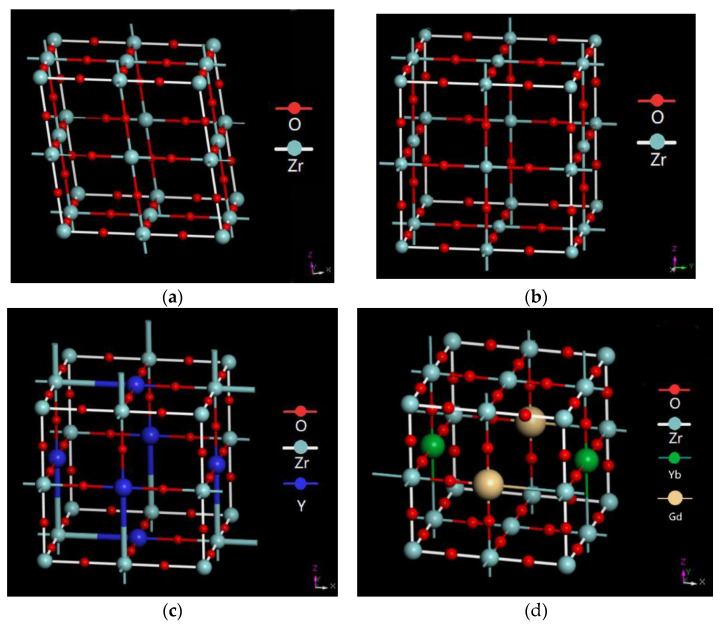
Crystalline structures of the ceramic: (**a**) M-ZrO_2_; (**b**) T-ZrO_2_; (**c**) T-8YSZ; (**d**) T-GYYZO.

Figure 9 shows the crystal structures of the ceramics including m-zirconia, t-zirconia, t-yttria stabilized zirconia (t-YSZ), and t-Gd_2_O_3_-Yb_2_O3-Y_2_O_3_-ZrO_2_ (t-GYYZO). Figure 9a shows the crystal structure of m-zirconia. Once t-zirconia transformed to m-zirconia, which was called a martensitic phase transformation, a large lattice shear and volume expansion effect would form. Therefore, in order to ensure the service life of the thermal barrier coatings, it was necessary to find a way to stabilize zirconia in a tetragonal structure. The monoclinic structure of zirconia had seven coordination numbers between zirconium and oxygen ions. Oxygen ions were sandwiched on one side of the tetrahedral coordination and the other side of the triangle coordination. When Y_2_O_3_ was added as a stabilizer to ZrO_2_, Y^3+^ would replace one Zr^4+^ in the original monoclinic phase and introduced 1/2 of the oxygen vacancy to form a metastable tetragonal structure [39], as shown in Figure 9c. With the increase of the stabilizer Y_2_O_3_, the thermal conductivity of the material could be reduced significantly. Meanwhile, it would lead to the transformation of YSZ to a cubic phase. Both Gd and Yb were similar to Y, which had trivalent ions. Meanwhile, their oxides were more stable than Y_2_O_3_. 

Therefore, stabilizers of Gd_2_O_3_ and Yb_2_O_3_ instead of Y_2_O_3_ could maintain the stability of the t-phase accompanied with reducing the thermal conductivity of the material. When Gd_2_O_3_ and Yb_2_O_3_ were added into YSZ, Gd^3+^ and Yb^3+^ would play the same role as Y^3+^, replacing the original monoclinic phase, which would improve the performance of YSZ without changing its tetragonal structure as shown in Figure 9d.

Table 7 shows the lattice parameters of the unit cells and the space group of the phases calculated through the CrystalMaker software^®^. M-ZrO_2_ had a monoclinic structure with space group P2_1_, the lattice parameters of a (0.5168 nm), b (0.5232 nm) and c (0.5319 nm) and β = 99.194°. T-ZrO_2_ had a tetragonal structure with space group P4m_2_, the lattice parameters of a (0.5120 nm), b (0.5120 nm) and c (0.5250 nm) and β = 90°. T-8YSZ had a tetragonal structure with space group P4_2_, the lattice parameters of a (0.3657 nm), b (0.3657 nm) and c (0.5303 nm) and β = 90°. T-GYYZO had a tetragonal structure with space group P4_2_, the lattice parameters of a (0.3638 nm), b (0.3638 nm) and c (0.5169 nm) and β = 90°. As compared to T-8YSZ, T-ZrO_2_ and M-ZrO_2_, T-GYYZO had the smallest lattice parameters of the unit cell, which would take the most structural stabilization of tetragonal structure in zirconia.

In this work, 5 wt% Yb_2_O_3_ and 5 wt%, 10 wt%, 15 wt% Gd_2_O_3_ were added into 8YSZ powder as thermal barrier coating materials, and sintered at 1650 °C for 6 h, 12 h, 24 h. The densification of the 8YSZ bulk and GYYZO bulk with 5 wt%, 10 wt%, 15 wt% Gd_2_O_3_ reached 96.89%, 96.58%, 96.47%, and 96.22% after sintering at 1650 °C for 24 h. With the addition of Gd_2_O_3_ and Yb_2_O_3_, the whole densification degree reduced a little in the same sintering conditions. TBCs prepared with GYYZO ceramic would have better sintering resistance than 8YSZ, which would enrich the application of TBCs at a high temperature. At present, the related properties of some ceramics used in thermal barrier coatings are shown in Table 8. The best properties of 8YSZ bulk exhibited the hardness of 13 GPa, the elastic modulus of 230 GPa, the fracture toughness of 5.1 MPa·m^0.5^, thermal conductivity and linear expansion coefficient are 1.85 W/(m·k) at 1000 °C and 10 × 10^−6^ /K at 1100 °C, respectively [19,31,32,35,37]. The 10YbSZ bulk stabilized by Yb_2_O_3_ had the hardness of 12.3 GPa, the elastic modulus of 210 GPa, thermal conductivity and linear expansion coefficient are 1.8 W/(m·k) and 10.7 × 10^−6^/K, respectively, at 1100 °C [40,41]. The 6GdSZ bulk stabilized by Gd_2_O_3_ had the hardness of 10 GPa, the elastic modulus of 200 GPa, thermal conductivity and linear expansion coefficient are 1.5 W/(m·k) and 11.5×10^−6^/K, respectively, at 1100 °C [41,42]. The performance of the GYYZO bulk materials increased with the increase of Gd_2_O_3_. With the increase of Gd_2_O_3_ addition, the hardness, elastic modulus, and fracture toughness of the GYYZO bulk increased and the thermal conductivity and thermal expansion coefficient of the GYYZO bulk decreased. In addition, 15 wt% Gd_2_O_3_-GYYZO bulk sintered at 1650 °C for 24 h had the highest hardness, elastic modulus, and fracture toughness of 15.61 GPa, 306.88 GPa, 7.822 MPa·m^0.5^ and the lowest thermal conductivity and thermal expansion coefficient of 1.04 W/(m·k) and 7.89 × 10^−6^/°C at 1100 °C, respectively. 

Stabilizers of Gd_2_O_3_ and Yb_2_O_3_ instead of Y_2_O_3_ could not only maintain the stability of the t-phase of ZrO_2_ but also reduce the thermal conductivity of the GYYZO composite material, which were analyzed in crystal structure and proved experimentally in this work. Meanwhile, GYYZO would provide application possibilities for thermal barrier coatings at higher temperatures.

## 5. Conclusions

(1). The densification of the 8YSZ bulk and GYYZO bulk with 15 wt% Gd_2_O_3_ reached 96.89% and 96.22% sintered at 1650 °C for 24 h.

(2). With the increase of Gd_2_O_3_ addition, the hardness, elastic modulus, and fracture toughness of the GYYZO bulk increased and the thermal conductivity and thermal expansion coefficient of the GYYZO bulk decreased.

(3). GYYZO bulk with 15 wt% Gd_2_O_3_ sintered at 1650 °C for 24 h had the highest hardness, elastic modulus, and fracture toughness of 15.61 GPa, 306.88 GPa, 7.822 MPa·m^0.5^, and the lowest thermal conductivity and thermal expansion coefficient of 1.04 W/(m·k) and 7.89 × 10^−6^/°C at 1100 °C, respectively.

(4). The addition of Gd_2_O_3_ into YSZ could not only effectively reduce the thermal conductivity but also improve the mechanical properties, which would improve the thermal barrier coatings’ performances further.

## Figures and Tables

**Table 1:** Powders mixing parameters through ball milling.

Processing	Parameters
Powders Ball to powder weight ratio	8YSZ, Yb_2_O_3_, Gd_2_O_3_ 10:1
Grinding ball Wetting agent	Agate ball 1 wt% sodium stearate
Rotation speed	120 rpm
Time	20 h

**Table 2:** Isostatic pressing and high temperature sintering parameters.

Processing	Parameters
Pressure	200 MPa
Holding time	5 min
Sintering temperature	1650 °C
Sintering time	6 h, 12 h, 24 h

**Table 3:** The porosities of the sintered bulks through image processing.

Bulks	8YSZ	5 wt% Gd_2_O_3_-GYYZO	10 wt% Gd_2_O_3_-GYYZO	15 wt% Gd_2_O_3_-GYYZO
1650 °C-6 h	12.33%	13.59%	14.07%	14.64%
1650 °C-12 h	6.50%	6.94%	7.04%	7.21%
1650 °C-24 h	3.11%	3.41%	3.52%	3.76%

**Table 4:** The densities of the sintered 8YSZ and GYYZO bulks.

Bulks	8YSZ (g/cm^3^)	5 wt% Gd_2_O_3_-GYYZO (g/cm^3^)	10 wt% Gd_2_O_3_-GYYZO (g/cm^3^)	15 wt% Gd_2_O_3_-GYYZO (g/cm^3^)
1650 °C-6 h	5.012	5.203	5.231	5.254
1650 °C-12 h	5.346	5.603	5.660	5.712
1650 °C-24 h	5.540	5.816	5.873	5.923

**Table 5:** The densification of the sintered bulks.

Bulks	8YSZ	5 wt% Gd_2_O_3_-GYYZO	10 wt% Gd_2_O_3_-GYYZO	15 wt% Gd_2_O_3_-GYYZO
1650 °C-6 h	87.65%	86.39%	85.92%	85.35%
1650 °C-12 h	93.49%	93.04%	92.96%	92.78%
1650 °C-24 h	96.89%	96.58%	96.47%	96.22%

**Table 6:** Fracture toughness of the sintered bulks/MPa·m^0.5^.

Bulks	8YSZ	5 wt% Gd_2_O_3_-GYYZO	10 wt% Gd_2_O_3_-GYYZO	15 wt% Gd_2_O_3_-GYYZO
1650 °C-6 h	5.936	6.048	6.163	7.025
1650 °C-12 h	6.127	6.388	6.615	7.364
1650 °C-24 h	6.391	6.725	7.127	7.822

**Table 7:** Lattice parameters of the unit cells and the space group of the phases in the ceramics.

Crystal Phase	Lattice (Å)	Space Group	Wykoff Coordinates	Angle
M-ZrO_2_	a = 5.168 b = 5.232 c = 5.319	P2_1_	Zr (0.276,0.041,0.208) O (0.070,0.336,0.314) O (0.442,0.755,0.479)	β = 99.194°
T-ZrO_2_	a = b = 5.120 c = 5.250	P4m2	Zr (0.750,0.250,0.750) O (0.750,0.750,0.942)	β = 90°
T-8YSZ	a = b = 3.657 c = 5.303	P4_2_	Zr (0.750,0.250,0.750) O (0.750,0.750,0.942) Y (0.250,0.750,0.750)	β = 90°
T-GYYZO	a = b = 3.638 c = 5.169	P4_2_	Zr (0.750,0.250,0.750) O (0.750,0.750,0.942) Y (0.250,0.750,0.750) Yb (0.750,0.750,0.250) Gd (0.250,0.250,0.750)	β = 90°

**Table 8:** Properties of ceramics used in thermal barrier coatings.

Bulks	Hardness (GPa)	Elastic Modulus (GPa)	Fracture Toughness (MPa·m^0.5^)	Thermal Conductivity W/(m·k)	Thermal Expansion Coefficient (at 1100 °C)
8YSZ	13 [31]	230 [35]	5.1 [32]	1.85 (at 1000 °C) [19]	10 × 10^−^^6^/K [3,7]
10YbSZ	12.3 [41]	210 [40]	——	1.8 (at 1100 °C) [41]	10.7 × 10^−6^/K [41]
6GdSZ	10 [41]	200 [41]	——	1.5 (at 1100 °C) [42]	11.5 × 10^−6^/K [42]
15 wt%Gd_2_O_3_-GYYZO	15.61	306.88	7.822	1.04 (at 1100 °C)	7.89 × 10^−^^6^/K

## Data Availability

Not applicable.
